# 

**Published:** 1894-07

**Authors:** 


Vol. XLVIII. JULY, 1894. No. 7.

### THE DENTAL REGISTER

A MONTHLY JOURNAL.

DEVOTED TO THE INTERESTS OF THE PROFESSION.

---

EDITED BY

J. TAFT. D.D.S.

ASSOCIATE EDITORS,

WILL. H. WHITSLAR, D.D.S. N S. HOFF, D.D.S.

---

PUBLISHED BY

SAM'L A. CROCKER & CO.,

Successors to Spencer & Crocker,

OHIO DENTAL AND SURGICAL DEPOT,

Nos. 117, 119, 121 WEST FIFTH STREET, CINCINNATI, OHIO.

**TERMS:—$2.00 per Annum, in Advance. Single Copies, 25 cts.**

